# The contribution of childhood circumstances, current circumstances and health behaviour to educational health differences in early adulthood

**DOI:** 10.1186/1471-2458-9-164

**Published:** 2009-05-29

**Authors:** Laura Kestilä, Tuija Martelin, Ossi Rahkonen, Tommi Härkänen, Seppo Koskinen

**Affiliations:** 1 National Institute for Health and Welfare (THL), Division of Welfare and Health Policies, Living conditions, Health and Wellbeing Unit, Helsinki, Finland; 2 University of Helsinki, Department of Public Health, Helsinki, Finland

## Abstract

**Background:**

The life course approach emphasises the contribution of circumstances in childhood and youth to adult health inequalities. However, there is still a lot to know of the contribution of living conditions in childhood and youth to adult health inequalities and how later environmental and behavioural factors are connected with the effects of earlier circumstances. This study aims to assess a) how much childhood circumstances, current circumstances and health behaviour contribute to educational health differences and b) to which extent the effect of childhood circumstances on educational health differences is shared with the effects of later living conditions and health behaviour in young adults.

**Methods:**

The data derived from the Health 2000 Survey represent the Finnish young adults aged 18–29 in 2000. The analyses were carried out on 68% (n = 1282) of the sample (N = 1894). The cross-sectional data based on interviews and questionnaires include retrospective information on childhood circumstances. The outcome measure was *poor self-rated health*.

**Results:**

Poor self-rated health was much more common among subjects with primary education only than among those in the highest educational category (OR 4.69, 95% CI 2.63 to 8.62). Childhood circumstances contributed substantially (24%) to the health differences between these educational groups. Nearly two thirds (63%) of this contribution was shared with behavioural factors adopted by early adulthood, and 17% with current circumstances. Health behaviours, smoking especially, were strongly contributed to educational health differences.

**Conclusion:**

To develop means for avoiding undesirable trajectories along which poor health and health differences develop, it is necessary to understand the pathways to health inequalities and know how to improve the living conditions of families with children.

## Background

Socio-economic health inequalities [1,2] seem to emerge rapidly when heading into adulthood: they are small or non-existent in childhood and adolescence [3-6], but marked already at early middle age [7-9]. Health differences related to socioeconomic position (SEP) are generated by various factors and mechanisms [10]. Higher SEP may promote better living and healthier working conditions [11-13], as well as healthier lifestyle, attitudes and choices [14] and is usually associated with physically less strenuous and psychosocially more rewarding work and better housing conditions than lower SEP. Moreover, compared with persons with a low SEP, those with a high SEP tend to smoke less [15-17], drink less alcohol [18,19], be physically more active [20,21], have healthier nutrition habits [22] and less likely be obese [23,24]. However, health itself can have an influence on SEP, those with poorer health [25] and health-damaging life-style [26] may end with a low SEP in adulthood.

The differences in health by SEP may arise from circumstances in early life which affect one's education, living conditions, health behaviour and, consequently, health. Social environment in childhood is associated with one's youth trajectories (i.e. educational career, family formation and employment paths) [27] as well as with health behaviour (smoking [28,29], heavy alcohol use [30,31], obesity [32] and physical inactivity [33], among other factors) and health [34,35]. The life course approach [36-38] suggests that long-term exposure to physical risks or adverse social and economic circumstances or concurrent adverse circumstances due to unfavourable living conditions in earlier life may lead to poor health [39,40]. We found in our previous studies that poor childhood circumstances were associated with poor SRH in early adulthood [41] and that poor childhood circumstances were associated with smoking [42], obesity [43] and heavy drinking [44], which are known to be more prevalent in lower educated groups and generate poor health.

Although there are several theories explaining health inequalities, their relative significance is still poorly understood [45]. There is still a lot to know about the contribution of childhood living conditions and youth to adult health inequalities and about how later environmental and behavioural factors are connected with the effects of earlier circumstances. An important phase of the life-course is early adulthood when health behaviours are largely established and health inequalities emerge. However, few studies [5,46-48] aim to explain the health inequalities in early adulthood from this perspective.

The aim of this study is to assess a) how much childhood circumstances, current circumstances and health behaviour contribute to the educational differences in poor self-rated health (SRH) and b) to which extent the effect of childhood circumstances on educational health differences is shared with the effects of later living conditions and health behaviour in young adults. Self-rated health is used as a general indicator of health, as it is a strong predictor of functional capacity [49], future health problems [50], as well as mortality [51].

### A simplified model of the expected associations

**Figure 1:**
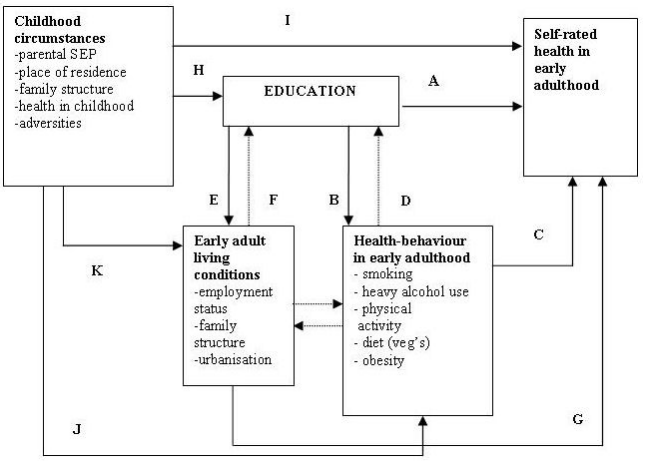
**Simplified model of the associations between childhood circumstances, education and other adult living conditions, health behaviour and health as operationalised in this study**.

A simplified model of the potential associations is presented in Figure 1. This study examines which factors contribute to the association between education and health (A). Health behaviours may mediate the association between education and health: education may promote healthier behavioural patterns (B), which in turn affect health (C). However, the opposite causal order between education and health behaviours is also plausible. Health-damaging behaviours adopted early in adolescence may partly select people to different educational positions (D), and thus explain part of the educational health differences. In the same way, early adult living conditions associated with both education (E and F) and health (G) may explain or mediate part of the association between education and health. Childhood circumstances are taken into account as possible explanatory factors potentially affecting both the respondent's education (H) and health (I). As childhood circumstances are assumed to affect health behaviour (J) and living conditions in early adulthood (K), a part of the contribution of childhood circumstances to educational health differences may be shared with that of the latter two categories of factors.

## Methods

### Participants

This study is based on a nationally representative sample of 1894 young adults in Finland aged 18–29 years. The data were collected in 2000–2001 as part of the Health 2000 Survey [52], using two-stage cluster sampling. The information was obtained with standardised structured computer-aided interviews (CAPI) and self-administered questionnaires to be returned later by mail. The participation rate in the interview was 79%. Questions concerning childhood adversities were asked in the questionnaire, which 85% of the interviewees answered. Thus, the analyses were carried out on 68% of the sample (n = 1282).

### Measures

*Self-rated health (SRH) *was based on the question "In general, would you say your health is..." with five response alternatives ranging from good to poor. Participants reporting 'average', 'quite poor' or 'poor' health were classified as having "poor SRH" (10%).

#### Childhood circumstances

*Parental education *was based on the participants' responses concerning their mother's and father's basic and vocational education (Table 1). The educational level of the parent whose educational level was the highest of the two was chosen to indicate parental education. *Family structure *was based on the question "when starting school (i.e. when you were about 7 years old), did you live...?" with four possible response alternatives "at home with both your parents", "with only one parent", "with relatives such as grandparents" and "in an orphanage or other institution", of which the last two were combined. *Urbanisation level of childhood residence *was categorised into "urban", "semi-urban" and "rural" [53]. Those living "abroad" were categorised into a separate group (n = 20).

**Table 1:** Distribution (%) of childhood and current circumstances and health behaviours by level of education and their associations (OR) with poor self-rated health in women and men aged 18–29 years in Finland. N = 1 282.

	**LEVEL OF EDUCATION**					**POOR SELF-RATED HEALTH**		
**EXPLANATORY FACTOR**	All	High	Middle	Primary				
	%	%	%	%	p^a^	OR	p^b^	p^c^
**Gender**								
Women	53	61	45	47		1.00		
Men	47	39	55	53	0.000	1.15	0.477	
**Age**								
18–23	61	57	65	60		1.00		
24–29	39	43	35	40	0.032	1.17	0.434	0.166
**CHILDHOOD CIRCUMSTANCES**								
								
**Parental education**								
Secondary	25	37	16	5		1.00		
Intermediate	24	27	21	21		2.01**		
Primary and some vocational	30	22	35	43		1.42		
Primary only	18	11	25	22		1.84*		
Don't know or did not have parents	3	2	2	8	0.000	1.64	0.149	0.866
**Childhood family structure**								
Two parents	92	93	93	81		1.00		
One parent	8	7	7	15		2.24**		
Other	0.6	0.5	0.2	3	0.000	3.14	0.008	0.053*
**Childhood residence**								
Urban municipalities	54	58	48	52		1.00		
Semi-urban municipalities	18	17	20	17		0.73		
Rural municipalities	27	23	31	28		1.24		
Abroad	1	1	1	2	0.039	0.37	0.239	0.134
								
**Childhood adversities (yes)**								
Long-term financial problems	17	16	17	23	0.235	2.09**	0.001	0.602
Parental regular unemployment	11	8	15	11	0.006	1.31	0.349	0.981
Parental divorce	20	17	21	37	0.000	1.65**	0.022	0.505
Serious conflicts within the family	24	25	23	28	0.484	2.42**	0.000	0.476
Parental mental health problem	8	7	7	10	0.664	1.80*	0.064	0.152
Parental alcohol problem	20	18	21	30	0.026	1.66**	0.026	0.113
Own serious or chronic illness	4	3	3	6	0.328	4.80**	0.000	0.250
Parental serious illness or disability	14	12	15	20	0.075	2.55**	0.000	0.854
Being bullied at school	25	22	25	35	0.024	2.89**	0.000	0.377
								
**CURRENT CIRCUMSTANCES**								
								
**Main activity**								
Full-time of part-time employed	60	58	64	48		1.00		
Student	22	33	13	1		1.07		
Unemployed or laid off	8	3	10	26		2.89**		
Other	10	5	13	25	0.000	1.52	0.001	0.449
**Current family structure**								
Married or cohabiting	53	57	48	51		1.00		
Living alone	26	32	21	16		1.32		
Living with own parents or other	21	11	30	32	0.000	1.20	0.460	0.254
**Current residence**								
Big city	44	53	33	44		1.00		
Urban or semi-urban	39	36	44	32		0.67*		
Rural	17	11	22	24	0.000	0.65	0.108	0.048**
**Having children (yes)**	21	15	25	35	0.000	1.16	0.527	0.137
								
**HEALTH BEHAVIOUR**								
								
**Daily smoking**	27	18	32	52	0.000	2.32**	0.000	0.204
**Heavy drinking**	6	4	6	25	0.000	2.63**	0.001	0.808
**Obesity**	7	5	8	11	0.035	2.69**	0.002	0.514
**Leisure time physical activity**								
4 times or more a week	29	31	26	35		1.00		
1–3 times a week	54	57	52	42		1.09		
Less than once a week	17	12	21	23	0.000	3.18**	0.000	0.278
**Use of vegetables**								
6–7 days a week	52	62	44	34		1.00		
3–5 days a week	27	24	30	31		1.16		
Less than 3 days a week	20	13	26	35	0.000	1.80**	0.031	0.335

p < 0.1, **p < 0.05

a Significance of the difference between educational level and explanatory factor, Chi2-test

b Significance of the difference between explanatory factor and poor self-rated health, Wald test p-value within the group

c Significance of the interaction between explanatory factor and gender, Wald test p-value

d Interaction gender*childhood family structure p = 0.053. The association between family structure and poor SRH is stronger in men than in women, p = 0.000 and p = 0.102, respectively

Childhood adversities were based on a pattern of eleven questions starting "when you think about your growth years, i.e. before you were aged 16, ...?". The effect of long-term financial problems, parental regular unemployment, parental divorce, serious conflicts within the family, parental mental health problems, parental alcohol problems, own serious or chronic illness, parental serious illness or disability and being bullied at school was tested (parental mental health problems as well as alcohol problems combined both the mother's and father's respective problems).

#### Current circumstances

The respondent's own education was based on the highest completed level of education. Because many respondents were still studying (21% of the original participants (n = 1505)), the measure for those studying was based on the expected level of education after the completion of their studies. A three-class variable was constructed: primary (only primary level education), medium (secondary level or lower degree tertiary) and high (higher degree level tertiary or higher). The respondent's main economic activity was categorised as "full-time or part-time employed", "student", "unemployed or laid off" and "other", and the urbanisation level of current residence as "big cities" (10 biggest cities by population), "other urban and semi-urban", and "rural" municipalities [53]. Current family structure was categorised as "married or cohabiting", "single" and "living with parent(s) or other(s)". The respondents were also classified as either with or without children.

#### Health behaviour and obesity

*Daily smokers *were defined as respondents who had smoked regularly for at least one year and most recently today or yesterday [42,54]. *Heavy drinking *was classified as consumption of ≥ 140 (women) and ≥ 280 (men) grams of pure alcohol per week [55,56]. It was based on information about both the frequency of drinking and the consumed quantity at a time for different types of alcohol during the past 12 months. *Body Mass Index (BMI) *was calculated from self-reported weight and height (weight/height^2^), and obese persons were defined as persons with BMI 30 kg/m^2 ^or over [57]. *Leisure-time physical activity *was based on the question "How often do you exercise in your leisure time so that you are at least slightly out of breath and sweating?" with three response alternatives: "less than once", "1–3 times" and "4+ times" a week. Considering health, the recommendation for this kind of exercise is at least three times a week, lasting 20–60 minutes at a time [58]. *Use of vegetables *was based on the question "How often have you eaten vegetables and roots (not potatoes), during the past week (7 days) as such, grated or in fresh salads?" Three classes were constructed: "6–7", "3–5" and "≤ 2" times a week. The use of vegetables is suggested to be one of the indicators for healthy nutrition [59].

### Statistical analysis

**Figure 2:**
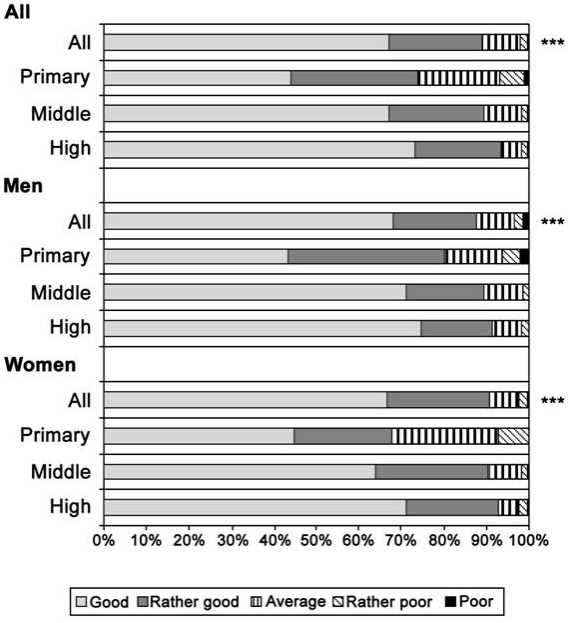
**Distribution (%) of self-rated health by level of education in all young adults and separately among women and men**. Statistical difference between educational groups, ***p < 0.001.

The associations between poor SRH and childhood as well as current determinants were analysed using logistic regression analysis and cross-tabulation. The sampling design and non-response [52] were accounted for by using the survey procedures of the STATA software [60] and poststratification weights [61]. Results are presented in terms of odds ratios (OR) with 95% confidence intervals (CI) and percentages (%). First, we present the distribution of SRH by level of education (Figure 2). Secondly, the associations between potential explanatory factors and poor SRH and education are presented (Table 1). We use p-values (Chi2 and Wald test) to present the significance of these associations. Significance of the interactions between gender and each explanatory factor was tested (Table 1). For further modelling we chose explanatory factors that are associated with both poor SRH and education on the significance level p < 0.25. This significance level was used because our aim was to study the effect of several potential factors on health inequalities. All included factors have a theoretical connection with health inequalities.

The results on the contribution of explanatory factors to the educational differences in poor SRH were calculated using multivariate logistic regression analysis, producing OR:s for the dependent variable (Table 2). In Model I, the educational differences in poor SRH were adjusted for age and gender. Additional explanatory factors were added (first one at a time and then in groups) to obtain Models II-V. The observed reduction in the strength of association between education and SRH from Model I to the subsequent models represents the contribution of the explanatory factor(s) to educational differences between educational groups. Percentage reduction was calculated as in previous studies [10,62-64]: (OR_(base model)_) - (OR_(base model+intermediate factor(s))_)/(OR_(base model)_- 1) × 100%.

**Table 2:** Age and gender-adjusted educational differences in poor SRH, adjusting for childhood circumstances, current circumstances and health behaviour. Odds ratios (OR) with 95% CI:s and reduction in percentages (%).

**MODEL I:** **Age+gender+educational level**	**High**	**Middle**	**Primary**	**% reduction in OR**^a^	
				
	**1.00**	**1.56 [0.98–2.48]**	**4.69 [2.63–8.32]**	**Middle**	**Primary**
**I+CHILDHOOD CIRCUMSTANCE(S)**					

Parental education	1.00	1.55 [0.97–2.46]	4.74 [2.62–8.57]	2	-1
Childhood family structure	1.00	1.55 [0.98–2.48]	4.33 [2.41–7.78]	2^b^	10^b^
Urbansation level of childhood residence	1.00	1.57 [0.98–2.52]	4.79 [2.69–8.53]	-2	-3
Financial difficulties in childhood family	1.00	1.54 [0.97–2.45]	4.54 [2.53–8.15]	4	4
Parental divorce	1.00	1.51 [0.94–2.40]	4.27 [2.38–7.68]	9	11
Parental alcohol problem	1.00	1.49 [0.93–2.37]	4.48 [2.54–7.90]	13	6
Parental serious illness of disability	1.00	1.47 [0.92–2.38]	4.50 [2.53–7.99]	16	5
Being bullied at school	1.00	1.47 [0.92–2.33]	4.27 [2.36–7.75]	16	11
					
All childhood circumstances (Model II)	1.00	1.46 [0.91–2.36]	3.80 [2.00–7.23]	18	24
					
**I+CURRENT CIRCUMSTANCE(S)**					

Main activity	1.00	1.58 [0.96–2.60]	4.19 [2.18–8.08]	-4	14
Urbanisation level of current residence	1.00	1.73 [1.09–2.75]	5.04 [2.83–8.99]	-30^c^	-10^c^
					
All current circumstances (Model III)	1.00	1.73 [1.06–2.83]	4.41 [2.29–8.49]	**-30**	**8**
					
**I+HEALTH BEHAVIOUR(S)**					

Daily smoking	1.00	1.41 [0.88–2.26]	3.80 [2.06–6.99]	27	24
Heavy drinking	1.00	1.52 [0.96–2.43]	4.13 [2.29–7.46]	7	15
Obesity	1.00	1.51 [0.94–2.43]	4.29 [2.40–7.66]	9	11
Physical activity	1.00	1.39 [0.87–2.23]	4.41 [2.50–7.76]	30	8
Use of vegetables	1.00	1.49 [0.92–2.39]	4.32 [2.43–7.70]	13	10
					
All health behaviours (Model IV)	1.00	1.22 [0.74–2.01]	3.00 [1.60–5.61]	**61**	**46**

**ALL FACTORS ADJUSTED (Model V)**	1.00	1.36 [0.79–2.33]	2.61 [1.17–5.83]	**36**	**56**

a Reduction % was calculated: [(OR_(model I)_-OR_(model I+intermediated factor(s)_)/(OR_(model 1)_-1)]

b Interaction between gender and family structure was p = 0.059. Childhood family structure reduced the educational differences only in men (8% between the high and the middle and 13% between the high and the primary educational categories whereas in women the differences increased by 9% and 19%, respectively

Finally, we analysed to what extent childhood circumstances, current circumstances and health behaviour had shared effects on the educational health differences (Table 3). The shared effects of two sets of explanatory variables were calculated by first summing up the reductions in the strength of association between education and SRH observed when including the two sets of variables separately in the age-adjusted model. From this sum we subtracted the reduction observed when including both sets of explanatory factors simultaneously in the age-adjusted model. The result of this subtraction represents the shared effect of the two sets of variables. The proportion of the effect of childhood circumstances shared by current circumstances and/or health behaviour was estimated as the ratio between the shared effect and the effect of childhood circumstances alone.

**Table 3:** Age- and gender-adjusted differences in poor SRH between the high and the primary educational category^a^, adjusting for childhood circumstances, current circumstances and behavioural factors.

**Adjusted factors**	**Educational level**	**High**	**Primary**	
		***OR [95 CI]***	**% reduction in OR**^b^	**Proportion shared %**
Base model^c^	1.00	4.69 [2.63–8.32]		
Childhood circumstances	1.00	3.80 [2.00–7.23]	24	
Current circumstances	1.00	4.41 [2.29–8.49]	8	
Behavioural factors	1.00	3.00 [1.60–5.61]	46	
Childhood and current circumstances	1.00	3.64 [1.78–7.41]	28	
Childhood circumstances and behavioural factors	1.00	2.65 [1.72–2.02]	55	
All	1.00	2.61 [1.17–5.83]	56	
Shared effect of current and childhood circumstances (%)			(24+8)-28 = 4	
Shared effect of behavioural factors and childhood circumstances (%)			(24+46)-55 = 15	
Proportion of the effect of childhood circumstances shared with current circumstances (%)				4/24 = 17
Proportion of the effect of childhood circumstances shared with behavioural factors (%)				15/24 = 63

Odds ratios (OR) with 95% CI:s and percentage reduction (%). Shared effects of behavioural factors/current circumstances and childhood circumstances (%) and the proportion (%) of the effect of childhood circumstances shared with current circumstances/health behaviour.

a Reduction % and shared effects not caluculated for the middle educational category as it did not differ significantly from the reference category in the base model

b Reduction % was calculated: [(OR_(base model)_-OR_(base model+intermediated factors)_)/(OR_(base model)_-1)]

c Adjusted for age and gender

### Ethical considerations

The plans and protocols for the Health 2000 Survey have been submitted for approval to the relevant ethical committees. The application was reviewed by the National Public Health Institute's Ethical Committee in September 1999. Following changes in legislation, a more detailed project plan was submitted to the Ethical Committee for Research in Epidemiology and Public Health at the Hospital District of Helsinki and Uusimaa (HUS) in May 2000. At both stages, the plans received favourable opinions. All necessary permissions and informed consent of the subjects have been acquired when the data collection was conducted.

## Results

### Educational differences in poor SRH

Of young adults, 70% rated their health good, 20% reported it as rather good, 8% as average and 2% considered their health rather poor or poor (Figure 2). There was no statistically significant gender difference in SRH (p = 0.449). Of these test subjects, 48% belonged to the highest, 44% to the middle and 8% to the lowest educational category. There was a clear gradient in poor SRH according to the respondent's educational level in both genders: the lower the respondent's education the more likely it was to report average or poorer health (p < 0.001). In the highest educational category, 7% reported average or poorer health, while the corresponding percentages were 10% in the middle and 26% in the lowest educational category (Figure 2). The interaction between educational level and gender in the age-adjusted model was not statistically significant (p = 0.215). The results are presented for men and women together and the interactions between single explanatory factors and gender are reported if found significant.

### Associations of childhood circumstances, current circumstances and health behaviour with poor SRH and level of education

The respondents whose parents had a secondary degree education were the least likely to report poor SRH, and the higher the parental education the more likely it was for the respondent to have reached or to reach a higher education level as well (Table 1). Similarly, living in a single-parent family in childhood increased the risk of poor SRH and the risk of belonging to the lowest educational category. Urbanisation level of childhood residence had a strong association with the respondent's education but only a weak association with poor SRH.

Of childhood adversities, long-term financial problems (OR = 2.09), parental divorce (OR = 1.65), serious conflicts within the family (OR = 2.42), parental mental health problems (OR = 1.80), parental alcohol problems (OR = 1.66), own serious or chronic illness (OR = 4.80), parental serious illness or disability (OR = 2.55) and being bullied at school (OR = 2.89) predicted poor SRH. Parental regular unemployment was not associated with poor SRH. Serious conflicts within the family, parental mental health problems and the respondent's own serious or chronic illness were not associated with the respondent's own educational level, and these childhood adversities were therefore removed from further analysis. All the other childhood adversities which predicted SRH were also associated with the respondent's education at the p < 0.25 significance level, which was the inclusion criterion.

Unemployed and laid off respondents had the highest risk of poor SRH (OR = 2.89) and being in this group also associated with low educational level. Urbanisation level of current residence associated with both poor SRH and low education. The association between current residence and poor SRH was stronger among women than in men (p = 0.048). Current family structure and having children were associated with the respondent's education, but not with poor SRH. These factors were, thus, removed from the further analyses.

Daily smokers (OR = 2.32), heavy drinkers (OR = 2.63), physically inactive (OR = 3.18) and obese respondents (OR = 2.65), and those not eating vegetables frequently (OR = 1.80) were significantly more likely to report poor health than those in the reference categories. All health-behavioural factors were associated with the respondent's educational level. Those in the lowest educational category had the most detrimental health behaviours.

### Explanatory effects of childhood circumstances, current circumstances and health behaviour on educational health differences

The effect of parental education on the educational differences in poor SRH was negligible (Table 2). The effect of childhood family structure was stronger: it explained almost one tenth of the differences between the highest and the lowest educational category. However, adjusting for childhood family structure reduced the educational health differences only in men (see Table 2, footnote b). Parental divorce and being bullied at school reduced the OR for poor SRH in the lowest educational category by 11%. For the middle educational category, being bullied at school (16%), parental serious illness or disability (16%) and parental alcohol problem (13%) reduced the ORs the most. All childhood circumstances together explained almost one fifth (18%) of the difference in poor SRH between the highest and the middle educational category and one quarter (24%) of the difference between the highest and the lowest educational category.

Adjusting for the respondent's main activity attenuated the difference in poor SRH between the highest and the lowest educational category by 14%. Adjusting for the urbanisation level of current residence, on the other hand, accentuated the educational differences, but this was only seen in men (see Table 2, footnote c). Together these two factors explained 8% of the health differences between the highest and the lowest educational category.

Daily smoking (24%) and heavy drinking (15%) greatly reduced the differences in poor SRH between the highest and the lowest educational category. Also obesity (11%), use of vegetables (10%) and physical activity (8%) reduced the differences. Furthermore, almost one third of the health difference between the highest and the middle category was explained by physical activity (30%) and by daily smoking (27%). All these behavioural factors together explained almost a half (46%) of the difference in poor SRH between the highest and the lowest, and even more (61%) of the difference between the highest and the middle educational category.

### Current circumstances and health behaviour as potential mediators of childhood circumstances

The contribution of childhood circumstances to the difference in SRH between the highest and the lowest educational category was 24%. Nearly two thirds (63%) of this effect was shared with behavioural factors adopted by early adulthood, and 17% with current circumstances (Table 3).

## Discussion

We found a strong association between education and SRH in young adulthood: the lower the education the poorer the health. Our results strengthen the assumption that educational health differences in adulthood result from factors operating at different stages of the life course [48] as childhood social circumstances explained a substantial part of the educational differences in health in young adulthood. However, the effect of childhood circumstances was largely shared with health behaviours adopted by early adulthood. Health behaviours, smoking especially, were strongly associated with educational health differences.

### Educational health differences and explanatory factors

SEP differences in health and health behaviours seem to emerge rapidly when heading into adulthood [5,27], after a period of subtle differences in youth [6,65,66]. We found wide educational differences in poor SRH already in early adulthood. One possible explanation for the rapid emergence of health differences in early adulthood is that many behavioural and environmental determinants of health get established at this phase of life. However, it is also possible that the effects of early environment and life-course do not become apparent until this stage of life [5].

In our data, both childhood circumstances and health behaviour during adolescence and young adulthood contributed to the health inequalities. Together, all the childhood circumstances included in our analyses explained one fourth of the differences between the highest and the lowest educational groups in poor SRH. The strongest single explanatory factors were parental divorce and in men, living in a single-parent family in childhood. Corresponding results have been reported previously for mortality [27]. Also other childhood circumstances contributed to educational health differences: for example, having been bullied at school was a strong determinant. This may also affect later trajectories due to psychological mechanisms. In general, those suffering unpredictable home life in childhood seem to have an increased risk for poor health and lower education in their early adulthood.

Health behaviour of young adults explained a large part of educational health differences, which corresponds with findings concerning broader adult age groups [67-69]. It is worth noting that behavioural patterns are partly adopted before the final level of education has been determined and, if behavioural patterns acquired early in life affect the later educational track as has been suggested [70], behavioural patterns adopted at young age may be partly responsible for educational health differences. However, it is also evident that low level of education increases the risk of many health endangering behaviours, and behavioural factors may thus partly mediate the effect of education on health. Daily smoking made the largest contribution to educational health differences in our study, but also heavy drinking, nutrition (indicated by use of vegetables), obesity and physical activity played important roles. Although use of vegetables is an adequate proxy for a healthy diet [22], it encompasses only a part of healthy nutrition. However, a recent Finnish study showed that use of vegetables contributed strongly to educational differences in both cardiovascular and total mortality among adults [69]. The impact of obesity on health inequalities probably increases as people grow older.

Current circumstances explained some of the differences between educational groups. This was due to the effect of main activity, as low education and poor health were particularly common among unemployed respondents. This is in accordance with previous studies on the health of unemployed young adults [71,72]. Living conditions in early adulthood may in some cases affect both the level of education and health, and influence their development, but the opposite causal order may be more important: living conditions in early adulthood are likely to be partly determined by the level of education.

### Pathways to health differences

A childhood disadvantage affects both socioeconomic circumstances and health in adulthood through various processes, for example, a child's development, health behaviours and the associated educational and social trajectories [73]. According to our results, the effect of childhood circumstances on differences in SRH between the lowest and the highest educational category was largely shared by behavioural factors adopted in youth and early adulthood. Almost two-thirds of the effect of childhood circumstances was shared by behavioural factors adopted by early adulthood, daily smoking being the strongest single factor. Some of the effect was shared also with the respondent's current circumstances (17%). Other potential factors not covered in our study include psychosocial factors and working conditions [11-13], for example.

The results of our study can be interpreted to support the role of both material and behavioural mechanisms in the development of health inequalities. Education affects health behaviour, which in turn influences health, and our results are in accordance with this pathway. On the other hand, we cannot rule out the hypothesis that health-related selection leads to the observed socioeconomic health inequalities [25], as we could not measure health in childhood and adolescence in a comprehensive way. The respondents' retrospective self-reports on their own chronic or long-term illness, however, did not explain the educational differences in health at all (< 1%), and it was not associated with education in the first place (p = 0.328). This finding suggests that health-related selection does not explain health inequalities among young adults in Finland. Other selection mechanisms, however, may have a more important role. Early initiation of smoking or heavy drinking, for example, may have affected both educational trajectories, as suggested in previous studies [26,74], and health, but we were not able to analyse the importance of these pathways in our study. However, the possibility of reverse causality should be kept in mind.

Our results suggest that socioeconomic health differences are partly due to the fact that early social circumstances affect both educational achievements and health in adulthood. This is often defined as indirect selection [27], which refers to a situation where low SEP as such does not cause poor health, but, instead, low SEP and poor health are both caused by a third factor. Our results point to a pathway from childhood social circumstances to adult educational health differences through uneven distribution of health behaviour and unequal adult living conditions. However, childhood social circumstances appeared to affect educational health differences also through other mechanisms that remain unidentified in this study.

### Methodological considerations

The strengths of the study include a nationally representative sample with a high participation rate, the breadth of indicators of childhood circumstances and the potential to study their concordance with current circumstances and several indicators of health behaviour. A clear limitation is the cross-sectional and retrospective nature of the data. For instance, we could only approximate the age at which the respondents had been exposed to adversities during childhood, and the effects may depend on the age at which these adversities were experienced, although no moderating effects of age have been reported in a corresponding setting [75,76]. Furthermore, it is possible that current circumstances to some extent affect the retrospective perceptions of childhood conditions and problems [77-79]. In addition, it is possible that people use different criteria when reporting childhood problems. This is a problem if the criteria vary systematically according to the other variables used in this study; however, there is no reason to expect that. Underreporting of chronic conditions, for example, seems to be slightly more common among less educated persons than among those with a higher education [80]. There is no reason to believe that underreporting of childhood adversities would show an opposite pattern. If young adults with low education tend to underreport childhood adversities more often than respondents with a higher education, our result concerning the contribution of childhood circumstances to health inequalities in early adulthood is likely to be an underestimate.

Results based on retrospective reports should be interpreted with caution. Although cross-sectional data do not offer the same benefits as a longitudinal design, there are good reasons for arguing that cross-sectional design with retrospective inquiries can yield reliable information. First, earlier studies have reported good test-retest reliability [77] and suggested that epidemiologic studies can validly use retrospective data on childhood SEP to study its relation to adult health status [81]. Secondly, information on the childhood living conditions and health was collected as a part of a major survey and there was no particular emphasis on the data used here.

Left truncation due to migration and mortality can be problematic in retrospective studies, as the population at the measurement time is not the same as at the (hypothetical) baseline. The left truncation mechanism might depend on the factors under study, and therefore compromise the conclusions. In our case, however, mortality is low, because the study was limited to the age group of 18–29 years. Also immigration and emigration to/from Finland has been low, thus the population has been stable, and the retrospective design of the study is adequate.

SRH has been suggested to be a good and valid measure of health [82,83], particularly in early adulthood when clinical endpoints are uncommon [48]. However, it is possible that different social groups may report their health differently, although there is no strong evidence on that.

Our measure of the respondent's education could not take into account the fact that some subjects may have temporarily "finished" their education or would interrupt their current studies later. However, regarding poor health and health-damaging behaviour, persons who later continue their education may resemble those who already have a higher level of education. It is therefore possible that the effect of education would have been even stronger than we report if we had been able to predict the final level of education for all participants. The same consideration applies in a case where some young adult interrupts his/her on-going education.

As the participation rate was 68%, the proportion of non-participants would undermine the reliability and validity of the results, if the characteristics of these non-participants differed considerably from those of the participants. A non-participation analysis has been reported elsewhere [84], and these findings give no indication that variation in the participation rate would crucially affect the results of this study, considering that also the weight system for statistical analyses constructed for the data corrects a part of the errors. In addition, even if the prevalence of various factors within the participants differed from those of the non-participants, we would have no reason to expect that the associations would be different in them.

## Conclusion

There are educational differences in health already in early adulthood. Childhood social circumstances affect later health differences, and this effect is largely shared with the effect of health behaviours adopted by young adulthood. Our study showed that educational differences are developed throughout life. Understanding the reasons and pathways to health inequalities and improving the living conditions of families with children could prevent the unfortunate trajectories by which poor health and health differences are developed.

## Competing interests

The authors declare that they have no competing interests.

## Authors' contributions

LK participated in the design of the study, performed the statistical analyses and wrote the first draft of the manurcript. TM, OR, SK and TH participated in the design of the study, commented the draft versions of the manuscript and helped to improve it. All authors read and approved the final manuscript.

## Pre-publication history

The pre-publication history for this paper can be accessed here:



## References

[B1] Kunst AE, Bos V, Lahelma E, Bartley M, Lissau I, Regidor E, Mielck A, Cardano M, Dalstra JA, Geurts JJ (2005). Trends in socioeconomic inequalities in self-assessed health in 10 European countries. Int J Epidemiol.

[B2] Mackenbach JP, Bos V, Andersen O, Cardano M, Costa G, Harding S, Reid A, Hemstrom O, Valkonen T, Kunst AE (2003). Widening socioeconomic inequalities in mortality in six Western European countries. Int J Epidemiol.

[B3] Pensola TH, Valkonen T (2000). Mortality differences by parental social class from childhood to adulthood. J Epidemiol Community Health.

[B4] West P (1988). Inequalities? Social class differentials in health in British youth. Soc Sci Med.

[B5] Rahkonen O, Arber S, Lahelma E (1995). Health inequalities in early adulthood: a comparison of young men and women in Britain and Finland. Soc Sci Med.

[B6] West P, Sweeting H (2004). Evidence on equalisation in health in youth from the West of Scotland. Soc Sci Med.

[B7] Mackenbach JP, Kunst AE, Cavelaars AE, Groenhof F, Geurts JJ (1997). Socioeconomic inequalities in morbidity and mortality in western Europe. The EU Working Group on Socioeconomic Inequalities in Health. Lancet.

[B8] Pensola TH, Valkonen T (2002). Effect of parental social class, own education and social class on mortality among young men. Eur J Public Health.

[B9] Valkonen T, Martikainen P, Jalovaara M, Koskinen S, Martelin T, Mäkelä P (2000). Changes in socioeconomic inequalities in mortality during an economic boom and recession among middle-aged men and women in Finland. European Journal of Public Health.

[B10] van Oort FV, van Lenthe FJ, Mackenbach JP (2005). Material, psychosocial, and behavioural factors in the explanation of educational inequalities in mortality in The Netherlands. J Epidemiol Community Health.

[B11] Borg V, Kristensen TS (2000). Social class and self-rated health: can the gradient be explained by differences in life style or work environment?. Soc Sci Med.

[B12] Monden CW (2005). Current and lifetime exposure to working conditions. Do they explain educational differences in subjective health?. Soc Sci Med.

[B13] Schrijvers CT, Mheen HD van de, Stronks K, Mackenbach JP (1998). Socioeconomic inequalities in health in the working population: the contribution of working conditions. Int J Epidemiol.

[B14] Wardle J, Steptoe A (2003). Socioeconomic differences in attitudes and beliefs about healthy lifestyles. WardlJournal of Epidemiology and Community Health.

[B15] Laaksonen M, Rahkonen O, Karvonen S, Lahelma E (2005). Socioeconomic status and smoking: analysing inequalities with multiple indicators. Eur J Public Health.

[B16] Paavola M, Vartiainen E, Haukkala A (2004). Smoking from adolescence to adulthood: the effects of parental and own socioeconomic status. Eur J Public Health.

[B17] Power C, Graham H, Due P, Hallqvist J, Joung I, Kuh D, Lynch J (2005). The contribution of childhood and adult socioeconomic position to adult obesity and smoking behaviour: an international comparison. Int J Epidemiol.

[B18] Casswell S, Pledger M, Hooper R (2003). Socioeconomic status and drinking patterns in young adults. Addiction.

[B19] Droomers M, Schrijvers CT, Stronks K, Mheen D van de, Mackenbach JP (1999). Educational differences in excessive alcohol consumption: the role of psychosocial and material stressors. Prev Med.

[B20] Lindström M, Hanson BS, Östergren PO (2001). Socioeconomic differences in leisure-time physical activity: the role of social participation and social capital in shaping health related behaviour. Soc Sci Med.

[B21] Martinez-Gonzalez MA, Varo JJ, Santos JL, De Irala J, Gibney M, Kearney J, Martinez JA (2001). Prevalence of physical activity during leisure time in the European Union. Med Sci Sports Exerc.

[B22] Roos E, Talala K, Laaksonen M, Helakorpi S, Rahkonen O, Uutela A, Prättälä R (2008). Trends of socioeconomic differences in daily vegetable consumption, 1979–2002. Eur J Clin Nutr.

[B23] Ali SM, Lindström M (2006). Socioeconomic, psychosocial, behavioural, and psychological determinants of BMI among young women: differing patterns for underweight and overweight/obesity. Eur J Public Health.

[B24] Sobal J, Stunkard AJ (1989). Socioeconomic status and obesity: a review of the literature. Psychol Bull.

[B25] Haas SA (2006). Health selection and the process of social stratification: the effect of childhood health on socioeconomic attainment. J Health Soc Behav.

[B26] Koivusilta L, Rimpelä A, Rimpelä M (1998). Health related lifestyle in adolescence predicts adult educational level: a longitudinal study from Finland. J Epidemiol Community Health.

[B27] Pensola T (2004). From Past to Present: Effect of Lifecourse on Mortality and Social Class Differences in Mortality in Middle Adulthood.

[B28] Anda RF, Croft JB, Felitti VJ, Nordenberg D, Giles WH, Williamson DF, Giovino GA (1999). Adverse childhood experiences and smoking during adolescence and adulthood. JAMA.

[B29] Jefferis BJ, Power C, Graham H, Manor O (2004). Effects of childhood socioeconomic circumstances on persistent smoking. Am J Public Health.

[B30] Engels RC, Vermulst AA, Dubas JS, Bot SM, Gerris J (2005). Long-term effects of family functioning and child characteristics on problem drinking in young adulthood. Eur Addict Res.

[B31] Anda RF, Whitfield CL, Felitti VJ, Chapman D, Edwards VJ, Dube SR, Williamson DF (2002). Adverse childhood experiences, alcoholic parents, and later risk of alcoholism and depression. Psychiatr Serv.

[B32] Parsons TJ, Power C, Logan S, Summerbell CD (1999). Childhood predictors of adult obesity: a systematic review. Int J Obes Relat Metab Disord.

[B33] Huurre T, Aro H, Rahkonen O (2003). Well-being and health behaviour by parental socioeconomic status: a follow-up study of adolescents aged 16 until age 32 years. Soc Psychiatry Psychiatr Epidemiol.

[B34] Dube SR, Felitti VJ, Dong M, Giles WH, Anda RF (2003). The impact of adverse childhood experiences on health problems: evidence from four birth cohorts dating back to 1900. Prev Med.

[B35] Rahkonen O, Lahelma E, Huuhka M (1997). Past or present? Childhood living conditions and current socioeconomic status as determinants of adult health. Soc Sci Med.

[B36] Wadsworth ME (1997). Health inequalities in the life course perspective. Soc Sci Med.

[B37] Kuh D, Ben-Shlomo Y, eds (2004). A life course approach to chronic disease epidemiology.

[B38] Hertzman C, Power C, Matthews S, Manor O (2001). Using an interactive framework of society and lifecourse to explain self-rated health in early adulthood. Soc Sci Med.

[B39] Hemmingsson T, Lundberg I (2005). How far are socioeconomic differences in coronary heart disease hospitalization, all-cause mortality and cardiovascular mortality among adult Swedish males attributable to negative childhood circumstances and behaviour in adolescence?. Int J Epidemiol.

[B40] Galobardes B, Lynch JW, Smith GD (2008). Is the association between childhood socioeconomic circumstances and cause-specific mortality established? Update of a systematic review. J Epidemiol Community Health.

[B41] Kestilä L, Koskinen S, Martelin T, Rahkonen O, Pensola T, Aro H, Aromaa A (2006). Determinants of health in early adulthood: what is the role of parental education, childhood adversities and own education?. Eur J Public Health.

[B42] Kestilä L, Koskinen S, Martelin T, Rahkonen O, Pensola T, Pirkola S, Patja K, Aromaa A (2006). Influence of parental education, childhood adversities, and current living conditions on daily smoking in early adulthood. Eur J Public Health.

[B43] Kestilä L, Rahkonen O, Martelin T, Lahti-Koski M, Koskinen S (2009). Do childhood social circumstances affect overweight and obesity in early adulthood?. Scand J Public Health.

[B44] Kestilä L, Martelin T, Rahkonen O, Joutsenniemi K, Pirkola S, Poikolainen K, Koskinen S (2008). Childhood and current determinants of heavy drinking in early adulthood. Alcohol Alcohol.

[B45] Adler NE, Ostrove JM (1999). Socioeconomic status and health: what we know and what we don't. Ann N Y Acad Sci.

[B46] Power C (1991). Social and economic background and class inequalities in health among young adults. Soc Sci Med.

[B47] Power C, Matthews S (1997). Origins of health inequalities in a national population sample. Lancet.

[B48] Power C, Matthews S, Manor O (1998). Inequalities in self-rated health: explanations from different stages of life. Lancet.

[B49] Idler EL, Kasl SV (1995). Self-ratings of health: do they also predict change in functional ability?. J Gerontol B Psychol Sci Soc Sci.

[B50] Kaplan GA, Goldberg DE, Everson SA, Cohen RD, Salonen R, Tuomilehto J, Salonen J (1996). Perceived health status and morbidity and mortality: evidence from the Kuopio ischaemic heart disease risk factor study. Int J Epidemiol.

[B51] Idler EL, Benyamini Y (1997). Self-rated health and mortality: a review of twenty-seven community studies. J Health Soc Behav.

[B52] Aromaa A, Koskinen S, eds (2004). Health and Functional Capacity in Finland. Baseline Results of the Health 2000 Health Examination Survey.

[B53] Statistics Finland (2000). Municipalities and Regional Divisions Based on Municipalities.

[B54] Helakorpi S, Martelin T, Torppa J, Patja K, Vartiainen E, Uutela A (2004). Did Finland's Tobacco Control Act of 1976 have an impact on ever smoking? An examination based on male and female cohort trends. J Epidemiol Community Health.

[B55] Salaspuro M, Alho H, Autti-Rämö I, Eskola K, Holopainen A, Lönnqvist J, Mäkelä R, Poikolainen K, Roine R, Saarnio P (2005). Alkoholiongelmaisen hoito. Käypä hoito -suositus (in finnish). [Treatment of Alcohol Abuse. Finnish Current Care Guidelines]. Duodecim.

[B56] Di Castelnuovo A, Costanzo S, Bagnardi V, Donati MB, Iacoviello L, de Gaetano G (2006). Alcohol dosing and total mortality in men and women: an updated meta-analysis of 34 prospective studies. Arch Intern Med.

[B57] WHO (2000). Obesity: preventing and managing the global epidemic. Report of a WHO consultation.

[B58] Pate RR, Pratt M, Blair SN, Haskell WL, Macera CA, Bouchard C, Buchner D, Ettinger W, Heath GW, King AC (1995). Physical activity and public health. A recommendation from the Centers for Disease Control and Prevention and the American College of Sports Medicine. Jama.

[B59] Steingrimsdottir L, Ovesen L, Moreiras O, Jacob S (2002). Selection of relevant dietary indicators for health. Eur J Clin Nutr.

[B60] StataCorp (2005). Stata Statistical Software: Release 9/. College Station.

[B61] Lehtonen R, Pahkinen EJ (1994). Practical Methods for Design and Analysis of Complex Surveys.

[B62] Sainio P, Martelin T, Koskinen S, Heliövaara M (2007). Educational differences in mobility: the contribution of physical workload, obesity, smoking and chronic conditions. J Epidemiol Community Health.

[B63] Laaksonen M, Roos E, Rahkonen O, Martikainen P, Lahelma E (2005). Influence of material and behavioural factors on occupational class differences in health. J Epidemiol Community Health.

[B64] Stronks K, Mheen H van de, Looman C, Mackenbach JP (1996). Behavioural and structural factors in the explanation of socioeconomic inequalities in health: an empirical analysis. Sociology of Health and Illness.

[B65] Hagquist CE (2007). Health inequalities among adolescents: the impact of academic orientation and parents' education. Eur J Public Health.

[B66] Hanson MD, Chen E (2007). Socioeconomic status and health behaviors in adolescence: a review of the literature. J Behav Med.

[B67] Barger SD (2006). Do psychological characteristics explain socioeconomic stratification of self-rated health?. J Health Psychol.

[B68] Lynch JW, Kaplan GA, Salonen JT (1997). Why do poor people behave poorly? Variation in adult health behaviours and psychosocial characteristics by stages of the socioeconomic lifecourse. Soc Sci Med.

[B69] Laaksonen M, Talala K, Martelin T, Rahkonen O, Roos E, Helakorpi S, Laatikainen T, Prättälä R (2008). Health behaviours as explanations for educational level differences in cardiovascular and all-cause mortality: a follow-up of 60 000 men and women over 23 years. Eur J Public Health.

[B70] Koivusilta L, Rimpelä A, Vikat A (2003). Health behaviours and health in adolescence as predictors of educational level in adulthood: a follow-up study from Finland. Soc Sci Med.

[B71] Berth H, Forster P, Brahler E (2003). [Unemployment, job insecurity and their consequences for health in a sample of young adults]. Gesundheitswesen.

[B72] Ahs A, Westerling R (2006). Self-rated health in relation to employment status during periods of high and of low levels of unemployment. Eur J Public Health.

[B73] Graham H, Power C (2004). Childhood disadvantage and health inequalities: a framework for policy based on lifecourse research. Child Care Health Dev.

[B74] Koivusilta LK, Rimpelä AH, Rimpelä MK (1999). Health-related lifestyle in adolescence – origin of social class differences in health?. Health Educ Res.

[B75] Rodgers B, Power C, Hope S (1997). Parental divorce and adult psychological distress: evidence from a national birth cohort: a research note. J Child Psychol Psychiatry.

[B76] Sigle-Rushton W, Hobcraft J, Kiernan K (2005). Parental divorce and subsequent disadvantage: a cross-cohort comparison. Demography.

[B77] Dube SR, Williamson DF, Thompson T, Felitti VJ, Anda RF (2004). Assessing the reliability of retrospective reports of adverse childhood experiences among adult HMO members attending a primary care clinic. Child Abuse Negl.

[B78] Hardt J, Rutter M (2004). Validity of adult retrospective reports of adverse childhood experiences: review of the evidence. J Child Psychol Psychiatry.

[B79] O'Malley SS, Carey KB, Maisto SA (1986). Validity of young adults' reports of parental drinking practices. J Stud Alcohol.

[B80] Mackenbach JP, Looman CW, Meer JB van der (1996). Differences in the misreporting of chronic conditions, by level of education: the effect on inequalities in prevalence rates. Am J Public Health.

[B81] Krieger N, Okamoto A, Selby JV (1998). Adult female twins' recall of childhood social class and father's education: a validation study for public health research. Am J Epidemiol.

[B82] Martikainen P, Aromaa A, Heliövaara M, Klaukka T, Knekt P, Maatela J, Lahelma E (1999). Reliability of perceived health by sex and age. Soc Sci Med.

[B83] Krause NM, Jay GM (1994). What do global self-rated health items measure?. Med Care.

[B84] Koskinen S, Laiho J, Rinne S, Kuosmanen N, Alha P, Rissanen H, Martelin T, Heistaro S Participation, complementary data collection and other measures to increase participation rate. Methodology Report Health 2000 Survey.

